# Acetate ameliorates ovarian mitochondrial dysfunction in letrozole-induced polycystic ovarian syndrome rat model by improving mitofusin-2

**DOI:** 10.1186/s12576-024-00908-5

**Published:** 2024-04-01

**Authors:** Kehinde S. Olaniyi, Stephanie E. Areloegbe

**Affiliations:** Cardio/Endo-metabolic and Microbiome Research Unit, Department of Physiology, College of Medicine and Health Sciences, Afe Babalola University, P.M.B. 5454, Ado-Ekiti, 360101 Nigeria

**Keywords:** Androgen, HDAC2, Infertility, Mitochondrial dysfunction, Mitofusin-2, PCOS

## Abstract

Androgen excess and metabolic abnormality largely contribute to the pathogenesis of polycystic ovarian syndrome (PCOS), which primarily precipitates ovarian dysfunction and infertility in reproductive-age women. Impaired mitochondrial function and epigenetic alteration have been linked to the development of PCOS. However, it is unknown whether acetate would exert a therapeutic effect on ovarian mitochondrial dysfunction in PCOS. Herein, the study hypothesized that acetate reverses ovarian mitochondrial dysfunction in experimental PCOS rat model, possibly through modulation of mitofusin-2 (MFn2). Eight-week-old female Wistar rats were randomized into four groups (n = 5). Induction of PCOS was performed by 1 mg/kg letrozole (*p.o.*), administered for 21 days. Thereafter, the rats were treated with acetate (200 mg/kg; *p.o.*) for 6 weeks. The PCOS rats demonstrated androgen excess, multiple ovarian cysts, elevated anti-mullerian hormone and leptin and decreased SHBG, adiponectin and 17-β estradiol with corresponding increase in ovarian transforming growth factor-β1. Additionally, inflammation (tumor growth factor and nuclear factor-kB), elevated caspase-6, decreased hypoxia-inducible factor-1α and elevated histone deacetylase-2 (HDAC2) were observed in the ovaries of PCOS rats, while mitochondrial abnormality with evidence of decreased adenosine triphosphate synthase and MFn2 was observed in rats with PCOS. Treatment with acetate reversed the alterations. The present results collectively suggest that acetate ameliorates ovarian mitochondrial abnormality, a beneficial effect that is accompanied by MFn2 with consequent normalization of reproductive-endocrine profile and ovarian function. Perhaps, the present data provide hope for PCOS individuals that suffer infertility.

**Supplementary Information:**

The online version contains supplementary material available at 10.1186/s12576-024-00908-5.

## Introduction

Endocrine-metabolic disorder, particularly polycystic ovarian syndrome (PCOS) has a worldwide prevalence of 6–21%, and accounts for over 70% of infertility cases in reproductive aged women [7, 50]. Infertility has remained a global burden, which exposes victims to stigmatization, discrimination, ostracism, psychological distress, and marital discord or even divorce [46]. The social burden falls disproportionately on women than men, and it is increasingly associated with risk of chronic health conditions such as neurodegenerative and cardiovascular disease [28]. A previous survey reported that over 186 million women in low-resource setting countries suffer infertility while about 74 million women are victims of infertility in developed countries, suggesting that women of low resource-setting nations are mostly affected by infertility and this has been attributed to poor diagnosis of infertility-driven pathological disorders, including PCOS and inability to access assisted reproductive techniques and in such, many women in developing nations are abandoned to their childless destinies [25]. A number of studies have also reported that infertility often affects the life’s quality by causing economic distress and psychological trauma [11, 14].

Despite the decades of research, the PCOS etiology is unknown. Although the clinical manifestations have been identified to include polycystic ovaries, hyperandrogenism, and anovulation or oligomenorrhea as defined by Rotterdam criteria [17]. These have positively impacted physicians’ diagnosis of this condition over the years. However, some individuals living with PCOS are yet undiagnosed, which means its diagnosis remains suboptimal due to unclear etiology. The pathophysiological mechanism of PCOS is multifactorial. Nevertheless, a number of previous studies have documented metabolic dysregulation or insulin resistance (IR), which is an intrinsic factor in the manifestation of various endocrine and metabolic disorders such as obesity, dyslipidemia/non-alcoholic fatty liver disease (NAFLD) and type 2 diabetes mellitus (T2DM) [18, 55]. Other investigators have also demonstrated the involvement of excessive oxidative stress and inflammation in the pathophysiology of PCOS [4]. Similarly, recent studies from our laboratory revealed ovarian lipotoxicity and lipid peroxidation, contributing to ovarian morphological disruption and degenerated follicles in experimental animal model of PCOS [36, 38].

In addition, mitochondria are crucial in energy production, and mitochondrial dysfunction at the cellular level often affects systemic metabolic balance. Granulosa cell mitochondrial dysfunction in PCOS patients has been implicated as a contributing factor to reduce folliculogenesis, oocyte health and cell viability [48, 52, 59]. In animal model, excessive reactive oxygen species (ROS) with a declined mitochondrial function have been demonstrated to cause diminished ovarian reserve or premature ovarian insufficiency [1] that often contributes to ovarian failure [5]. Mitochondrial dysregulation often causes ROS formation, which have a pivotal impact on the biological/cellular functions at appropriate level. However, increased ROS production induces cellular damage/death, including mitochondrial-mediated apoptosis. Moreover, Eriksen et al. observed unaltered mitochondrial mass in insulin-resistant women with PCOS compared to healthy women [16]. Nevertheless, mitochondrial dysfunction accounts for several features of PCOS such as hyperandrogenism, metabolic dysregulation, inflammation and follicular disruption with or without oxidative stress [42]. Hence, the pathogenic role of mitochondrial abnormality in ovarian deficiency associated with PCOS needs further investigation, considering the increased prevalence of infertility in PCOS women.

Mitochondria compensate for a functional defect through fusion, which is mediated by mitofusin, particularly mitofusin-2, found in the outer mitochondrial membrane. Mitofusin-2 is a GTPase transmembrane protein that regulates the morphology of mitochondria and crucial in the development of metabolic disorder, including diabetes mellitus, insulin resistance, obesity, and related syndrome [12, 19]. It is also involved apoptotic cell regulation. Evidence exists that MFn2 expresses abundantly in the ovarian granular cell and theca cells, follicular fluid/corpus luteum and ovarian stroma in humans and animals [6]. However, earlier studies have demonstrated impaired follicular development in PCOS animals through down-regulation of MFn2 [30]. Similarly, reduced MFn2 expression has been reported to contribute to defective follicular maturation and female infertility, and this defect have been associated with mitochondrial dysfunction. Targeted deletion of MFn2 protein in the mitochondrion causes a decline in oocyte maturation and female infertility, with impaired oocyte granulosa cell communication, resulting in aberrant follicular development especially at the secondary follicle stage [8]. Recently, absence or reduction of mitofusin-2 was reported to cause cellular apoptosis with subsequent depletion of ovarian follicular reserve, phenotypes, and thus accelerated female reproductive aging. Therefore, it is important to further investigate the participation of mitofusin-2 in mitochondrial ovarian dysfunction associated with PCOS. This will possibly provide clinical relevance in the management of infertility in individuals with PCOS.

In addition to lifestyle modification, estrogen–progestin oral contraceptives and biguanide (metformin), are the first choice of therapy for women with PCOS [33, 57]. The first line of drug, oral contraceptives has been used to manage endocrine imbalance while treatment with biguanide has effectively reduced blood glucose, insulin resistance, and androgen level, thereby improving endocrinometabolic status of individuals with PCOS, although with side effect profile which causes poor compliance and discontinuation of these therapies [15, 43]. Emerging evidence exists that short-chain fatty acids (SCFAs) such as acetate, propionate and butyrate, derived from fermentation of indigestible high-fiber diet by gut microbiota, are immunometabolic modulators that regulate various physiological functions of cellular activities. Acetate remains the most abundant of SCFAs in circulation [9]. A number of studies have demonstrated that SCFAs, including acetate, has acted as cardioprotective, antioxidant, anti-lipolytic, insulin sensitizer in pathological disease model including metabolic and related syndrome [39, 21, 35, 37]. Nevertheless, the impact of acetate on ovarian mitochondrial dysfunction in PCOS is unknown. Albeit, the present study investigated the therapeutic impact of acetate on ovarian mitochondrial dysfunction in PCOS experimental rat model and the probable involvement of MFn2, a mitochondrial fusion protein.

## Materials and methods

### Experimental animals and grouping

The study was carried out and reported in accordance with the ARRIVE guidelines**.** Independent Ethical Review Board of Afe Babalola University (Ado-Ekiti, Nigeria) gave ethical approval with ABUADERC/09/2022, and the study was conducted in accordance with the guidelines of National Institutes of Health for the care and use of Laboratory Animals. Eight-week-old Wistar rats were purchased from the animal house of the institution. The rats were given free access to standard rat chow and tap water. Acclimatization of rats lasted for a week and rats were at least three consecutive regular estrous cycles with the same estrous stage, which was determined through vaginal smear. The rats were assigned randomly into four groups with *n* = 5 per group, namely control (CONT), sodium acetate (SATE), PCOS and PCOS + SATE groups. Rats were maintained in a colony under standard environmental conditions (22–26 °C of temperature), (50–60% of relative humidity), and 12-h dark/light cycle.

### Experimental induction and confirmation of PCOS

Polycystic ovarian syndrome was induced through letrozole (1 mg/kg; *p.o.*,) purchased from Sigma-Aldrich, St Louis, MI*,* and administered to rats in PCOS and PCOS + SATE groups for a period of 21 days as previously documented [27, 38]. Manifestation of PCOS was confirmed using Rotterdam criteria [17] with the determination of estrous cycle and testosterone level [36, 41]. Histology of the ovaries was performed at the end of the treatment with sodium acetate.

### Treatment of experimental animals

The control group received vehicle (distilled water, *p.o.*), SATE group received sodium acetate (200 mg/kg, *p.o.*,) obtained from Sigma-Aldrich, St Louis, MI, PCOS group received distilled water and PCOS + SATE group received sodium acetate and the treatment lasted for six weeks [10].

### Collection of samples

After the treatment and overnight fast, the rats were given anesthesia by injection of sodium pentobarbital (50 mg/kg, *i.p.*). Blood sample was collected via cardiac puncture into heparinized tube and centrifuged at 704 g for 5 min at room temperature. Plasma was stored at −80 °C until it was required for biochemical assays.

### Ovarian tissue homogenate preparation

The ovaries of each rat were isolated and weighed. Thereafter 100 mg of the tissue was removed carefully, minced and homogenized in 1 ml of 0.25 M sucrose/0.2 mM EDTA adjusted to pH 7.5 with Tris buffer. The homogenates of ovarian tissue were centrifuged at 4 °C for 12 min at 750 g. The supernatant fluid was collected and stored at −80 °C until it was required for biochemical assays.

### Isolation of mitochondria

Mitochondria isolation from the ovaries of the rats was performed by collecting the supernatant fluid of the tissue homogenate and centrifuged at 6700 g for 12 min. A resuspension of pellet was carried out and the 750 g and 6700 g centrifugation procedures were repeated twice. The mitochondria were then suspended in 5–10 ml of a solution containing 70 mM sucrose, 220 mM mannitol, 2 mM HEPES buffer, pH 7.4 and 1 mM EDTA.

### Biochemical analysis

#### Endocrine profile

Plasma concentrations of testosterone, anti-mullerian hormone (AMH), sex hormone binding globulin (SHBG), leptin, adiponectin and 17-β estradiol were determined using Rat ELISA kits produced by Calbiotech Inc. (Cordell Ct., USA) in adherence to manufacturer’s procedures.

#### Transforming growth factor β-1 (TGF-β1)

The concentration of TGF-β1 was determined from the ovarian tissue using rat ELISA kit obtained from Elabscience Biotechnology Inc. (Wuhan, Hubei, P.R.C., China), in compliance with manufacturer’s guidelines.

#### Inflammatory biomarkers

Concentrations of nuclear factor-kappaB (NF-kB) and tumor necrosis factor-α (TNFα) were determined from ovarian tissue using sandwich-ELISA principle with ELISA kits purchased from Elabscience Biotechnology Inc. (Wuhan, Hubei, P.R.C., China).

#### Caspase-6 and hypoxia inducible factor-1α

The level of caspase-6 was determined from the ovarian tissue using the principle of sandwich immunoassay employing ELISA kits produced by ELK Biotechnology Co. Ltd. (1312 17th Street #692 Denver, CO 80202 USA). Similarly, hypoxia inducible factor-1α (HIF-1α) was determined from ovarian tissue using ELISA kits purchased from Elabscience Biotechnology Inc. (Wuhan, Hubei, P.R.C., China).

#### ATP synthase, mitofusin-2 and histone deacetylase-2

The activities of adenosine triphosphate (ATP) synthase were measured from the mitochondria using standard spectrophotometric method with assay kits obtained from Elabscience Biotechnology Inc. (Wuhan, Hubei, P.R.C., China). The measurements were performed in strict adherence to the manufacturer’s guidelines. The level of ovarian mitochondrial MFn2 was measured using rat ELISA kits purchased from Wuhan Feiyue Biotechnology Co., Ltd (C6124, Building C6, No. 666, Gaoxin Avenue, Wuhan, Hubei, China). Ovarian tissue level of HDAC2 was determined using a sandwich immunoassay technique with rat specific ELISA kit produced by ELK Biotechnology Co. Ltd. (1312 17th Street #692 Denver, CO 80202 USA).

#### Histological and stereological evaluation of ovaries

For histological assessment using hematoxylin and eosin (H&E) staining technique, a section of the ovary was fixed in 10% formol saline overnight and thereafter dehydrated, embedded in paraffin, and sectioned at 5-μm thickness. The slides were prepared and OPTO-Edu industrial camera light microscope and a computer (Nikon, Japan) were used to examine and evaluate the slides. Stereological evaluation of ovarian follicles was determined as described in the previous studies [32, 38].

#### Data analysis

All data were expressed as means ± SD. GraphPad Prim software version 9 was used for data analysis and one-way ANOVA was employed for comparison of the group means. Post hoc analysis was done with Bonferroni’s test, and statistically significant difference was considered at p less than 0.05.

## Results

### Sodium acetate improves reproductive and metabolic profile as well as ovarian morphology in experimental rats that developed PCOS.

**Fig. 1:**
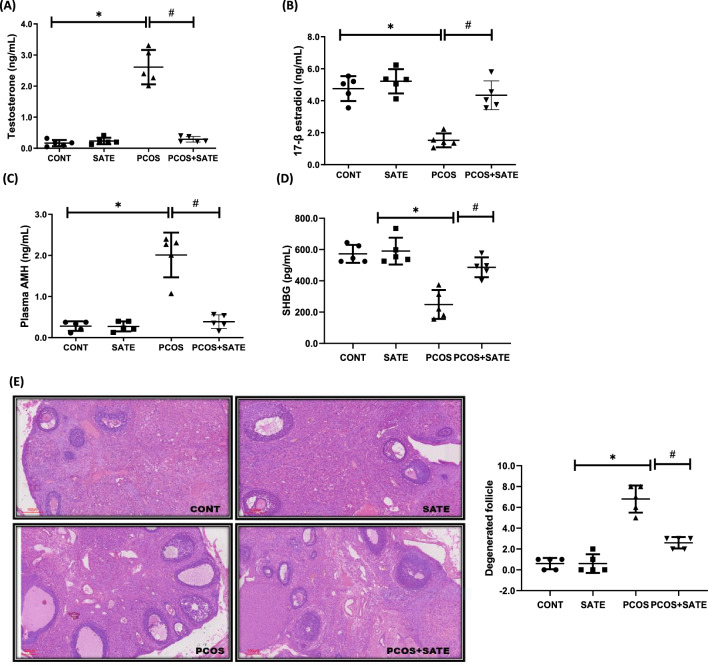
Sodium acetate’s impact on testosterone (**a**), 17β-estradiol (**b**), anti-mullerian hormone (**c**), sex hormone binding globulin (**d**), and ovarian histology (**e**) in experimental PCOS rats. Data are expressed with mean ± SD, *n* = 5. (*p < 0.05 vs control, #*p* < 0.05 vs PCOS). Polycystic ovarian syndrome (PCOS); control (CONT); sodium acetate (SATE); anti-mullerian hormone (AMH); sex hormone binding globulin (SHBG)

**Fig. 2:**
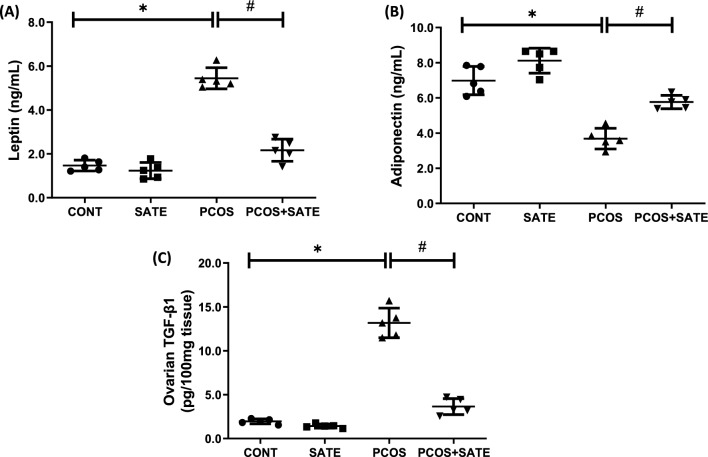
Sodium acetate’s impact on plasma leptin (**a**), adiponectin (**b**), and transforming growth factor-β1 (**c**) in experimental PCOS rats. Data are expressed with mean ± SD, *n* = 5. (*p < 0.05 vs control, #*p* < 0.05 vs PCOS). Polycystic ovarian syndrome (PCOS); control (CONT); sodium acetate (SATE); transforming growth factor-β1 (TGF-β1)

Experimental PCOS rats showed a significant increase (*p* < 0.05) in testosterone and AMH with a corresponding decrease in 17-β estradiol and SHBG compared with control, and administration of SATE significantly improved the alterations in reproductive hormonal profile as shown in PCOS + SATE compared with untreated PCOS group (Fig. 1a–d). In addition, ovarian histological evaluation revealed multiple cysts with antral follicles in PCOS group compared with normal ovarian morphology in control and improved ovarian morphology in PCOS + SATE group (Fig. 1e). Similarly, metabolic hormone, leptin significantly increased (p < 0.05), while adiponectin decreased in PCOS rats compared with control, and administration of SATE decreased leptin and increased adiponectin in PCOS + SATE group compared with untreated PCOS group (Fig. 2a–b). Ovarian transforming growth factor-β1 significantly increased in PCOS animals compared with control group, and this was decreased by sodium acetate administration as shown in PCOS + SATE group compared with untreated PCOS group (Fig. 2c).

### Sodium acetate decreases the levels of ovarian inflammatory and apoptotic biomarkers in experimental rats that developed PCOS

**Fig. 3:**
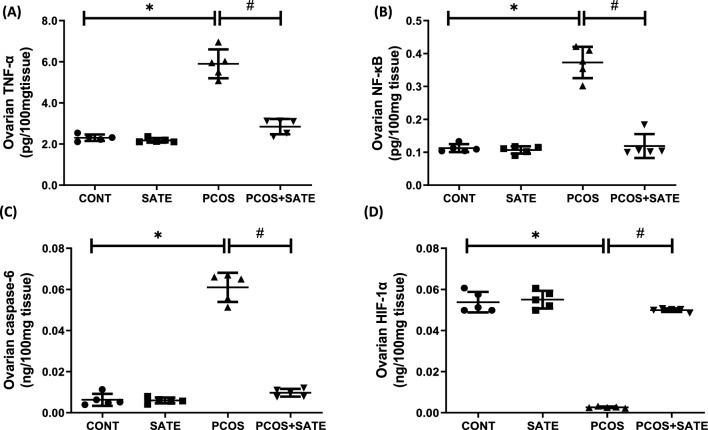
Sodium acetate’s impact on ovarian TNF-α (**a**), NF-kB (**b**), Caspase-6 (**c**), and HIF-1α (**d**) in experimental PCOS rats. Data are expressed with mean ± SD, *n* = 5. (*p < 0.05 vs control, #*p* < 0.05 vs PCOS). Polycystic ovarian syndrome (PCOS); Control (CONT); sodium acetate (SATE); tumor necrosis factor-α (TNF-α); nuclear factor-kappaB (NF-kB); hypoxia inducible factor-1α (HIF-1 α)

There was a significant increase (p < 0.05) in the ovarian levels of TNF-α, NF-kB and Caspase-6 with corresponding decrease in HIF-1α in experimental PCOS rats compared with control. Administration of SATE significantly decreased the levels of ovarian inflammatory biomarkers (TNF-α and NF-kB) and caspase-6 with an increase in HIF-1α level of PCOS + SATE group compared with untreated PCOS group (Fig. 3).

### Sodium acetate improves ovarian mitochondrial function and decreases ovarian level of HDAC2 in experimental rats that developed PCOS

**Fig. 4:**
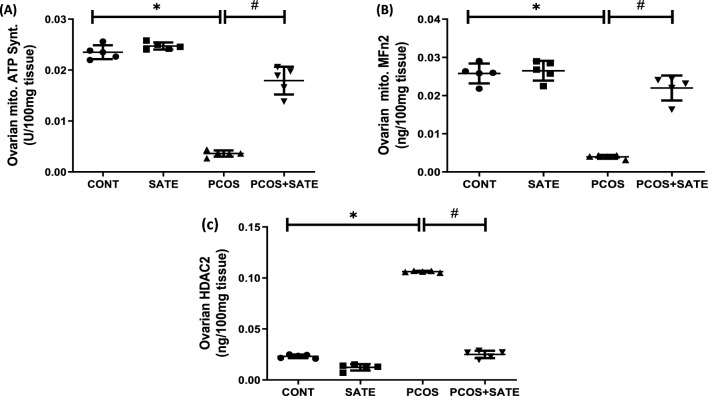
Sodium acetate’s impact on ovarian mitochondrial ATP synthase (**a**), mitofusin-2 (**b**), and HDAC2 (**C**) in experimental PCOS rats. Data are expressed with mean ± SD, *n* = 5. (*p < 0.05 vs control, #*p* < 0.05 vs PCOS). Polycystic ovarian syndrome (PCOS); control (CONT); sodium acetate (SATE); adenosine triphosphate (ATP); mitofusin-2 (MFn2); histone deacetylase-2 (HDAC2)

There was a significant reduction (*p* < 0.05) in mitochondrial ATP synthase and MFn2 in experimental PCOS rats compared with control. Nevertheless, administration of SATE significantly increased (*p* < 0.05) mitochondrial ATP synthase and MFn2 with a decrease in ovarian HDAC2 level in PCOS + SATE group compared with untreated PCOS group (Fig. 4).

## Discussion

The incidence of ovarian dysfunction and consequent infertility in PCOS women is recently on the increase, in both developed and developing countries [25]. This has led to socio-economic burden, mental stress, and divorce even among the young couples. This is unsurprisingly attributed to multifactorial pathophysiology and importantly, the unclear etiology of PCOS, necessitating further research on its pathophysiological mechanisms and etiopathology, with the hope of improving diagnosis, management, and treatment of PCOS. The key findings from the present study reveal the development of ovarian mitochondrial dysfunction in experimental model of PCOS. This is accompanied by suppressed level of MFn2. Interestingly, administration of acetate improves MFn2 level with normalization of ovarian mitochondrial function in experimental PCOS animals. In line with our previous studies [37, 40], PCOS animals showed excess body weight gain/ovarian mass, abnormal metabolic indices (fasting insulin, blood glucose and HOMA-IR) (Additional file 1: Figures S1, S2c), androgen excess, multiple ovarian cysts, elevated AMH and leptin and decreased SHBG, adiponectin and 17-β estradiol. These correspond with increased plasma and ovarian triglyceride (Additional file 1: Figures S2d and S3a), TGF-β1, and mitochondrial abnormality, with evidence of altered mitochondrial complex (ATP synthase) and subsequent ovarian apoptosis (caspase-6), which were associated with suppressed level of MFn2. However, the ovarian pathology with attendant mitochondrial dysfunction was reversed when treated with acetate.

In the present study, PCOS phenotypes and dyslipidemia were observed in animals that received letrozole, which were confirmed by increased testosterone (hyperandrogenism) and AMH levels, 17-β estradiol depletion and impaired androgen clearance (SHBG) with multiple ovarian cysts, as well as disrupted lipid profile compared with control animals. These observations are in agreement with previous studies, including our earlier studies [18, 36, 40, 56]. Testosterone–estrogen imbalance is a common endocrine factor in PCOS and earlier linked to impaired folliculogenesis [58, 59] that contributes to defective oocyte maturation. These altogether promote infertility in PCOS women [48]. In this study, the PCOS animals manifested excess body weight and ovarian mass, which suggest the presence of obesity and ovarian hypertrophy, as previously reported [19, 20]. The obese PCOS animals also demonstrated gluco-lipid dysregulation with evidence of IR and dyslipidemia, which are critical features of obesity, validating endocrine-metabolic derangement in animals with PCOS compared with control group. These observations are in consonance with investigations by Dumesic et al. and Wojciechowska et al. [13, 54].

Moreover, the metabolic hormones including leptin and adiponectin were significantly dysregulated in animals with PCOS when compared with control. Leptin and adiponectin are important adipocytokines which are essential for the regulation of energy and metabolic indices such as, insulin, glucose, and lipid via their insulin-sensitizing property [22, 49]. The present study revealed a possible alteration in adipose function, which might contribute to elevated level of leptin in untreated PCOS animals. Increase in circulating level of leptin can also be attributed to leptin resistance which is usually triggered by systemic and peripheral insulin resistance and these observations are similar to earlier studies including previous studies from our laboratory [29, 35]. Elevated circulating level of leptin is an indication of impaired metabolic function that is well-documented in PCOS individuals [26, 60], and is associated with suppressed level of plasma adiponectin. Hence, the present results suggest the exacerbation of metabolic disorder in PCOS. In addition, metabolic dysregulation or IR, often activates oxidative stress signals. In the present study, systemic metabolic dysregulation with clear manifestation of IR could trigger lipid influx into non-metabolic tissues, with ovarian tissue inclusive, contributing to elevated level of ovarian triglyceride in the untreated PCOS animals. This causes excessive ovarian lipid accumulation that results in ovarian lipotoxicity, which promotes ovarian oxidative stress (MDA) by depletion of antioxidant capacity as expressed with decreased level of antioxidant regulator (NrF2) in animals with PCOS compared with control group (Additional file 1: Figure S3b, c).

The present result also shows a significant increase in ovarian TGF β-1 in animals exposed to letrozole compared with control. Transforming growth factor is known to regulate cellular processes, including cell differentiation, proliferation and cell survival [47]. Nevertheless, its derangements have been implicated in the fetal origin of PCOS [3]. This possibly contributes to reproductive abnormalities in PCOS, and this is consistent with previous studies that demonstrate the contribution of TGF-β in PCOS pathogenesis [45]. Circulating levels of TGF-β have been previously correlated with androgen level in PCOS women, with evidence of higher expression levels of genes controlled by TGF β, promoting excessive collagen deposition. Therefore, the higher level of TGF-β, a master upstream regulator, possibly contributes to excess ovarian mass in animals exposed to letrozole. Additionally, ovarian lipotoxicity with TGF-β1-driven collagen production might affect ovarian response which is evident with elevated levels of TNF-α and NF-κB in animals exposed to letrozole compared with control. These are downstream and upstream proinflammatory mediators, respectively, that predispose ovarian tissue to apoptosis as expressed by increased level of Caspase-6, and decreased level of HIF-1α. Thus, contributing to disrupted mitochondrial function characterized by decreased level of mitochondrial complex (ATP synthase). The present findings suggest that animals exposed to letrozole significantly demonstrate impaired mitochondrial ovarian function, which contributes to ovarian dysfunction and perhaps the pathogenesis of PCOS.

Notably, the levels of MFn2, a mitochondrial fusion protein were significantly decreased in the ovaries of animals that received letrozole with corresponding decrease in ovarian mitochondrial ATP synthase, and increased levels of ovarian inflammatory mediators (TNF-α and NF-kB) as well as apoptotic markers (Caspase-6) and lipid peroxidation (MDA) with depletion of antioxidant regulator (NrF2) and HIF-1α compared with control group. These possibly disrupted ovarian morphology, characterized with multiple cysts and degenerated follicles as indicated histologically, thus leading to impaired ovarian function that manifested in elevated levels of androgen and AMH and a decline level of 17-β estradiol and SHBG. The present observations are similar to earlier studies, which attributed an aberrant follicular maturation and subsequent infertility to decreased expression of MFn2 [8, 30]. Similarly, targeted MFn2 deletion has been earlier documented as a critical contributor to impaired oocyte granulosa cell development that results in ovarian failure or deficiency [1]. Recent studies also demonstrated MFn2 reduction in ovarian apoptosis and follicular reserve depletion [2, 8]. Hence, the present findings suggest mitochondrial fusion protein MFn2 as a possible pathogenic link to ovarian mitochondrial dysfunction, thus contributing to PCOS pathogenesis. Therefore, modulation of MFn2 might probably provide a better therapeutic outcome to the management of PCOS. Similarly, a study by Zhu et al., demonstrated that HDAC activity down-regulates Mfn2 expression by deacetylation of Mfn2 promoter, leading to mitochondrial dysfunction and inhibition of HDAC prevented reduced Mfn2 expression [61].

Interestingly, administration of HDACi, acetate significantly increased ovarian mitochondrial fusion protein MFn2 with corresponding decrease in ovarian oxidative stress (MDA) and inflammation (NF-kB and TNF-α), attenuating ovarian apoptosis (Caspase-6 and HIF-1α) with a significant improvement in mitochondrial complex (mitochondrial ATP synthase) that is responsible for mitochondrial energy production in animals that received letrozole compared with control group. These subsequently improved ovarian morphology as demonstrated with normal cytoarchitecture and follicles with mild antral follicles, restoring ovarian function with evidence of increased 17-β estradiol and decreased AMH and testosterone in letrozole-induced PCOS animals in comparison to untreated PCOS group. These observations are similar to previous studies that demonstrated the beneficial impact of HDACi on ovarian function in metabolic related syndrome [34, 36], and in preclinical ovarian cancer [51]. Similarly, overexpression of MFn2 in the ovaries enhances endocrine function and promotes follicular maturation in rodents [24] as well as improves mitochondrial function [61]. Recently, Zhang et al. also supported that MFn2 is crucial in follicular and oocyte development and is involved in the regulation of ovarian follicular reserve, especially in reproductive ageing [58]. Therefore, the present findings suggest that HDACi, acetate restores ovarian mitochondrial function and consequent ovarian function in LET-induced PCOS animals, a beneficial effect that is accompanied by MFn2 enhancement. In addition, treatment with acetate significantly reduced ovarian mass, TG and TGF-β1 in animals that developed PCOS compared with untreated PCOS animals, attenuating ovarian lipotoxicity and excessive mass that triggered oxidative stress in untreated PCOS animals. Likewise, circulating levels of adiponectin/leptin were improved in PCOS + SATE with subsequent enhancement of insulin sensitivity as shown by a significant decrease in HOMA-IR, which is a surrogate marker of insulin resistance [23], suggesting that acetate mitigates metabolic dysregulation in rats that developed PCOS. These observations are similar to previous studies that demonstrate the beneficial impacts of SCFAs, including acetate in metabolic pathologies such as diabetes mellitus, PCOS among others [37, 44]. Overall, the present findings suggest the therapeutic benefit of HDACi, acetate on metabolic dysregulation and ovarian mitochondrial dysfunction in PCOS rat model, which is accompanied by mitochondrial fusion protein MFn2 enhancement. Although other inconclusive mechanisms through which acetate elicits its beneficial effects in different tissues have been suggested [21, 31, 53]. Nevertheless, the present data perhaps, provide clinical relevance toward efforts to reduce the burden of PCOS and subsequent infertility.

## Conclusion

The present results collectively suggest that acetate ameliorates ovarian mitochondrial abnormality, a beneficial effect that is accompanied by MFn2 with consequent normalization of reproductive-endocrine profile and ovarian function. Perhaps, the present data provide hope for PCOS individuals that suffer infertility.

### Supplementary Information

**Additional file 1: Figure S1.** Sodium acetate’s impact on body weight gain (a) and ovarian mass (b) in experimental PCOS rats. Data are expressed with mean ± SD, n = 5. (*p < *0.05 vs control, #p* < *0.05 vs PCOS).* Polycystic ovarian syndrome (PCOS); Control (CONT); Sodium acetate (SATE). **Figure S2.** Sodium acetate’s impact on fasting insulin (a), fasting blood glucose (b), HOMA-IR (c) and plasma triglyceride (d) in experimental PCOS rats. Data are expressed with mean ± SD, n = 5., n = 5. (*p < *0.05 vs control, #p* < *0.05 vs PCOS).* Polycystic ovarian syndrome (PCOS); Control (CONT); Sodium acetate (SATE); Homeostatic model of insulin resistance (HOMA-IR); Triglyceride (TG). **Figure S3.** Sodium acetate’s impact on ovarian triglyceride (a), MDA (b) and NrF2 (c) in experimental PCOS rats. Data are expressed with mean ± SD, n = 5. (*p < *0.05 vs control, #p* < *0.05 vs PCOS).* Polycystic ovarian syndrome (PCOS); Control (CONT); Sodium acetate (SATE); Triglyceride (TG); Malondialdehyde (MDA); Nuclear factor erythroid 2-related factor 2 (NrF2).

## Data Availability

The data supporting the present study will be made available from the corresponding author on request.
