# Supplementary material for: Acetate ameliorates ovarian mitochondrial dysfunction in letrozole-induced polycystic ovarian syndrome rat model by improving mitofusin-2
Source: J Physiol Sci. 2024 Apr 1;74:22. doi: 10.1186/s12576-024-00908-5 (PMC10983676; doi:10.1186/s12576-024-00908-5)
Supplement: Supplementary file 1 — Additional file 1: Figure S1. Sodium acetate’s impact on body weight gain (a) and ovarian mass (b) in experimental PCOS rats. Data are expressed with mean ± SD, n = 5. (*p < 0.05 vs control, #p < 0.05 vs PCOS). Polycystic ovarian syndrome (PCOS); Control (CONT); Sodium acetate (SATE). Figure S2. Sodium acetate’s impact on fasting insulin (a), fasting blood glucose (b), HOMA-IR (c) and plasma triglyceride (d) in experimental PCOS rats. Data are expressed with mean ± SD, n = 5., n = 5. (*p < 0.05 vs control, #p < 0.05 vs PCOS). Polycystic ovarian syndrome (PCOS); Control (CONT); Sodium acetate (SATE); Homeostatic model of insulin resistance (HOMA-IR); Triglyceride (TG). Figure S3. Sodium acetate’s impact on ovarian triglyceride (a), MDA (b) and NrF2 (c) in experimental PCOS rats. Data are expressed with mean ± SD, n = 5. (*p < 0.05 vs control, #p < 0.05 vs PCOS). Polycystic ovarian syndrome (PCOS); Control (CONT); Sodium acetate (SATE); Triglyceride (TG); Malondialdehyde (MDA); Nuclear factor erythroid 2-related factor 2 (NrF2). [file 12576_2024_908_MOESM1_ESM.docx]

**Additional file**


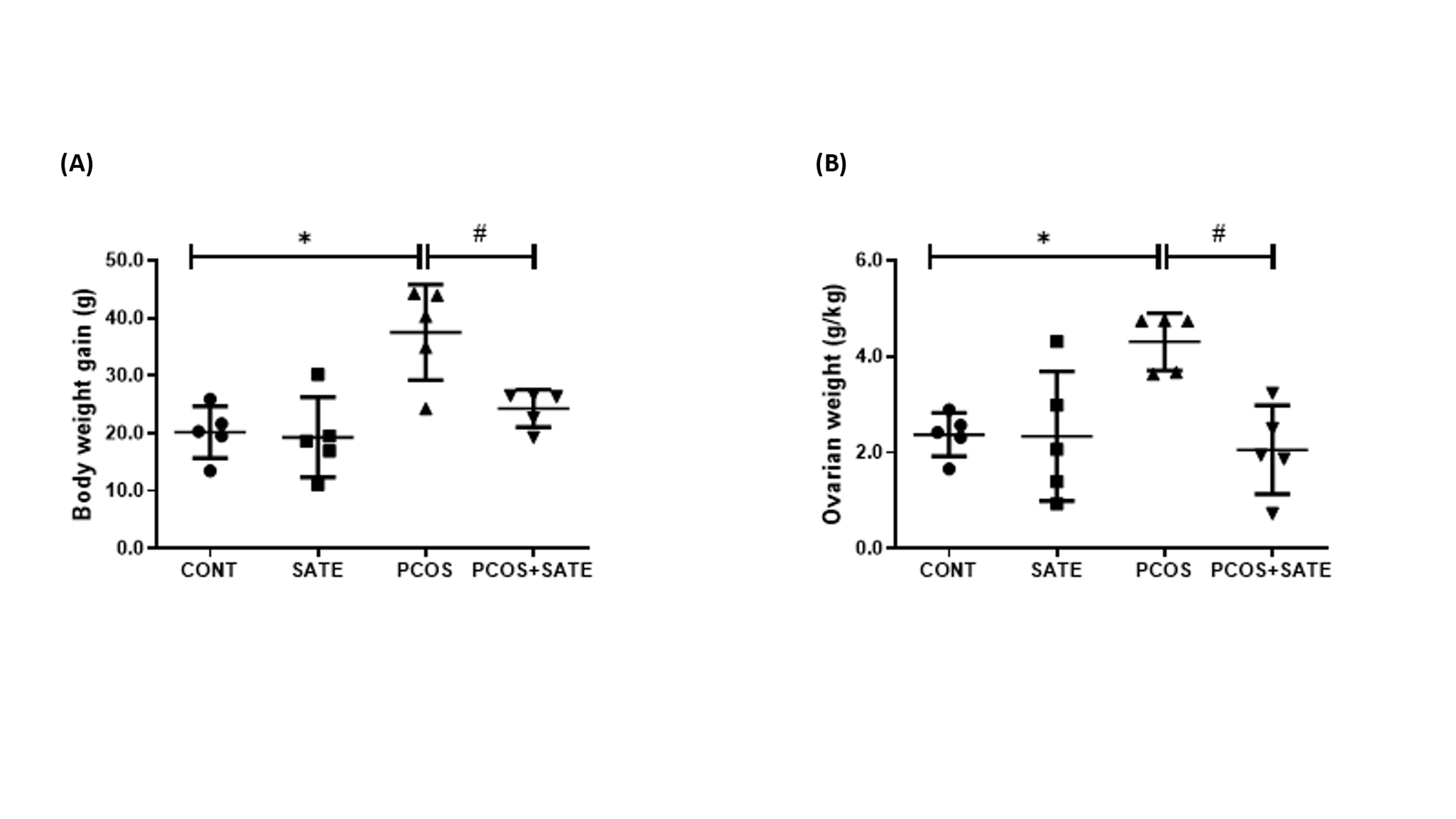


Figure 1. Sodium acetate’s impact on body weight gain (a) and ovarian mass (b) in experimental PCOS rats. Data are expressed with mean ±SD, n=5. *(*p<0.05 vs control, #p<0.05 vs PCOS).* Polycystic ovarian syndrome (PCOS); Control (CONT); Sodium acetate (SATE).


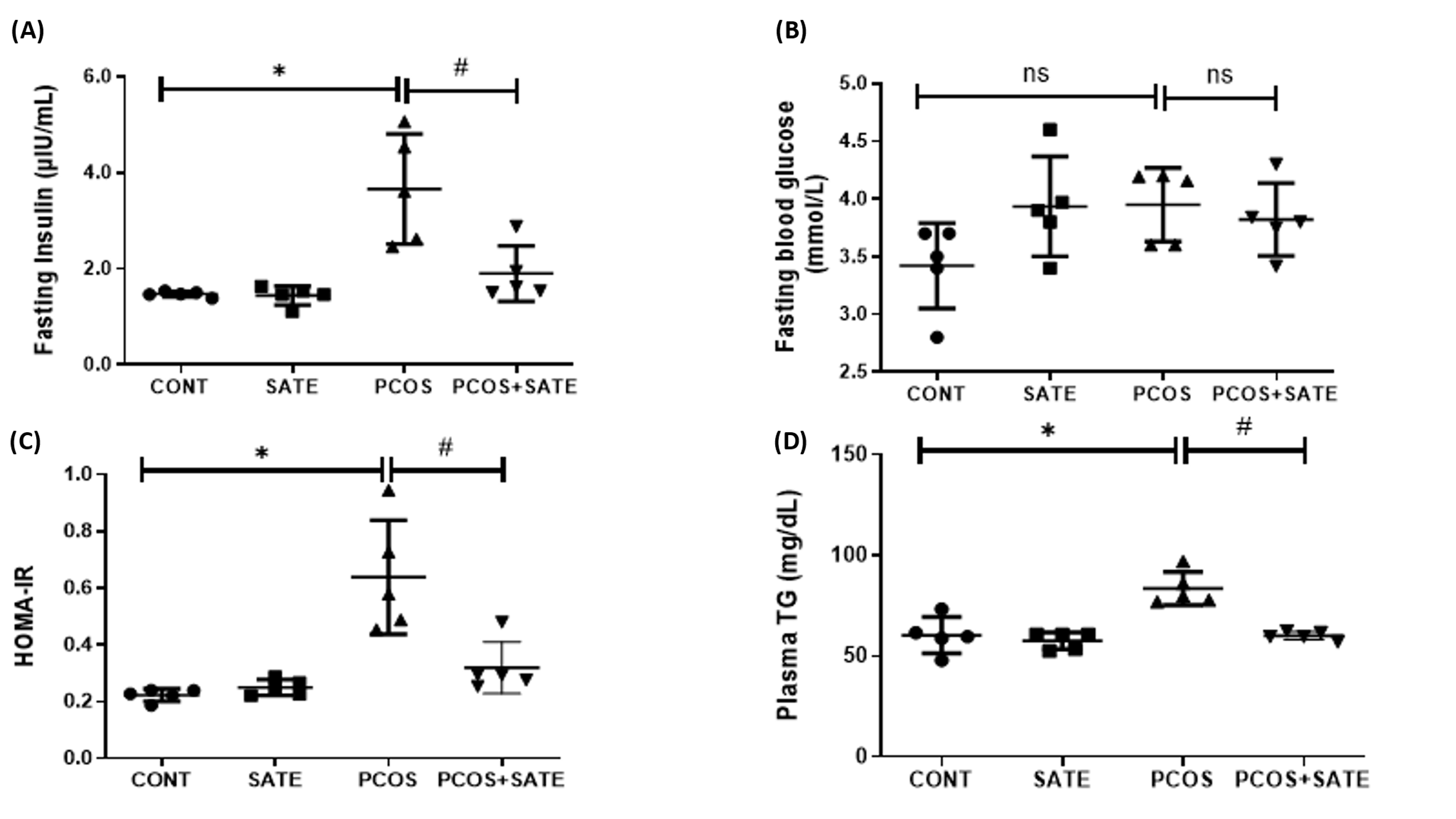


Figure 2. Sodium acetate’s impact on fasting insulin (a), fasting blood glucose (b), HOMA-IR (c) and plasma triglyceride (d) in experimental PCOS rats. Data are expressed with mean ±SD, n=5., n=5. *(*p<0.05 vs control, #p<0.05 vs PCOS).* Polycystic ovarian syndrome (PCOS); Control (CONT); Sodium acetate (SATE); Homeostatic model of insulin resistance (HOMA-IR); Triglyceride (TG).


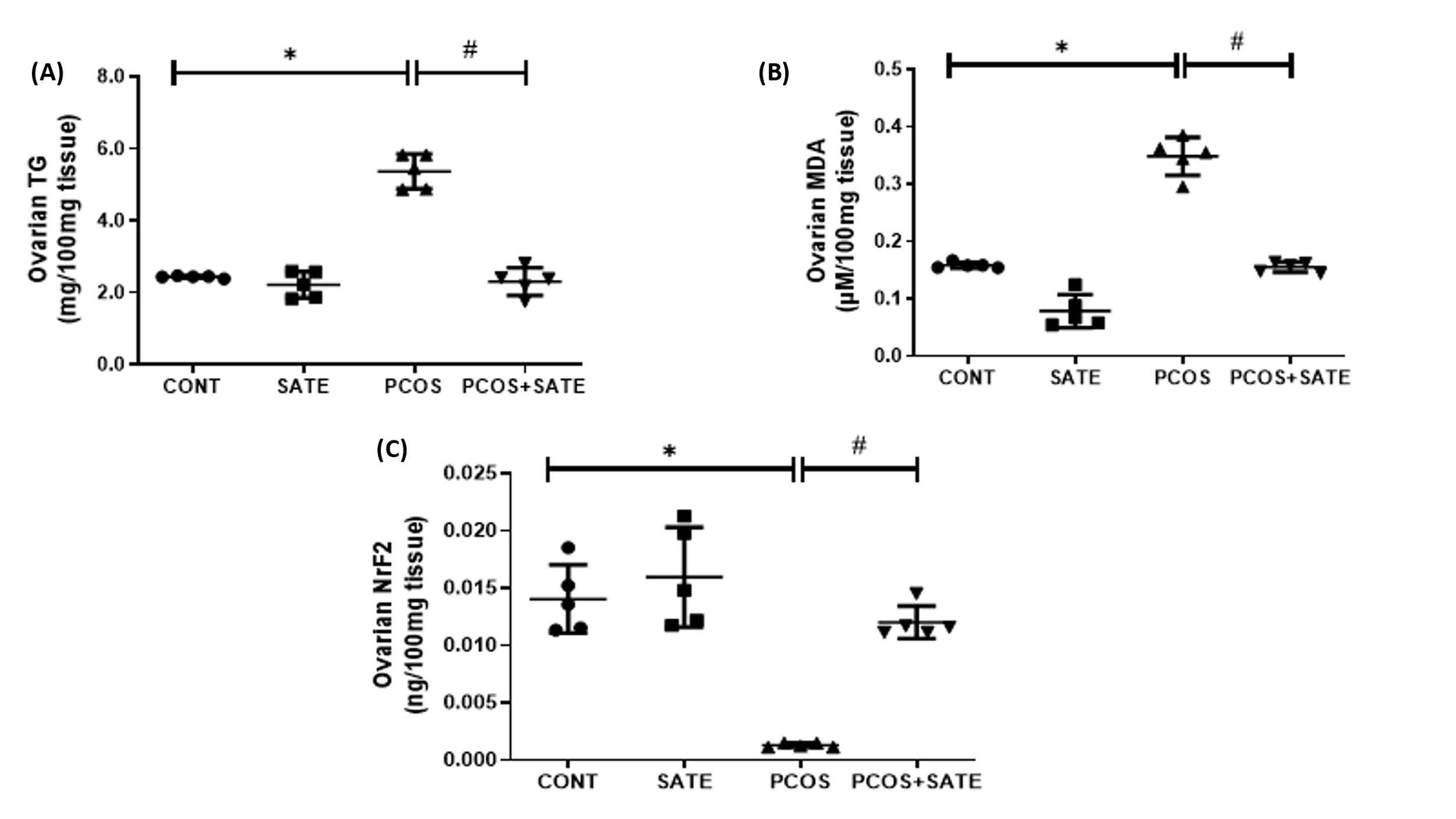


Figure 3. Sodium acetate’s impact on ovarian triglyceride (a), MDA (b) and NrF2 (c) in experimental PCOS rats. Data are expressed with mean ±SD, n=5. *(*p<0.05 vs control, #p<0.05 vs PCOS).* Polycystic ovarian syndrome (PCOS); Control (CONT); Sodium acetate (SATE); Triglyceride (TG); Malondialdehyde (MDA); Nuclear factor erythroid 2-related factor 2 (NrF2).
